# Cardiovascular Risk Management in Persons with Dementia

**DOI:** 10.3233/JAD-230019

**Published:** 2023-05-30

**Authors:** Charlotte Nijskens, Marieke Henstra, Hanneke Rhodius-Meester, Sevil Yasar, Eveline van Poelgeest, Mike Peters, Majon Muller

**Affiliations:** a Department of Internal Medicine, Amsterdam UMC location Vrije Universiteit Amsterdam, Geriatrics section, Amsterdam, The Netherlands; b Department of Neurology, Amsterdam UMC location Vrije Universiteit Amsterdam, Alzheimer Center Amsterdam, Amsterdam, The Netherlands; c Department of Geriatric Medicine, Oslo University Hospital, Oslo, Norway; d Department of Medicine, Division of Geriatric Medicine and Gerontology, Johns Hopkins School of Medicine, Baltimore, MD, USA; e Department of Internal and Geriatric Medicine, University Medical Center Utrecht, Utrecht, The Netherlands

**Keywords:** Alzheimer’s disease, cardiovascular diseases, cardiovascular risk, control, dementia, hypercholesterolemia, hypertension, prevention

## Abstract

The number of people living with dementia, such as Alzheimer’s disease, is increasing worldwide. Persons with dementia often have a high risk of atherosclerotic cardiovascular disease and they are therefore theoretically eligible for treatment of hypertension and hyperlipidemia. However, in this population, beneficial and harmful effects of cardiovascular risk management (CVRM) may be different compared to older persons without cognitive impairment. Current CVRM guidelines are based on trials from which persons with dementia were excluded. In this narrative review, we will discuss how current guidelines can be translated to persons with dementia and which aspects should be taken into account when treating hypertension and hyperlipidemia to prevent major adverse cardiovascular events (MACE). Survival time is significantly shorter in persons with dementia. We therefore suggest that since the main goal of CVRM is prevention of MACE, first of all, the patient’s life expectancy and treatment wishes should be evaluated. Risk assessment tools are to be used with care, as they tend to overestimate the 5- and 10-year risk of MACE and benefit from CVRM in the prevention of MACE in persons with dementia. When the clinician and patient have decided that treatment is initiated or intensified, patients should be closely monitored since they are at high risk for adverse drugs events and overtreatment due to the natural course of blood pressure in persons with dementia. In the event of intolerance or side effects, medication should be switched or withdrawn. For persons with dementia and limited life expectancy, deprescribing should be part of usual care.

## INTRODUCTION

According to current estimates, almost 55 million people are living with dementia worldwide. This number is expected to quadruple by 2050 due to population ageing and growth [1]. As a consequence, the burden of dementia on patients, families, health care, and long-term care systems is continuously growing, with dementia related health care costs representing 11–17% of all expected health spending by 2050 [2]. The clinical syndrome of dementia consists of several subtypes that are distinct in their etiology, clinical profile, and management, such as Alzheimer’s disease (AD) and vascular dementia (VaD). Mixed vascular and AD pathology are the most common pathologies underlying dementia in older persons and become more common when age increases, as both pathologies worsen over time [3]. AD and VaD share similar pathophysiological processes with cardiovascular disease (CVD), such as atherosclerosis, caused by common vascular factors like hypertension and hyperlipidemia [4]. Cardiovascular risk management (CVRM) can therefore be of interest in limiting the burden of atherosclerotic cardiovascular disease (ASCVD) and hence possibly cognitive decline in dementia.

From robust trial evidence in relatively healthy older persons without cognitive impairment, we know that treating hypertension and hyperlipidemia reduces the risk of myocardial infarction and stroke and might reduce the risk of cognitive decline [5–9]. In the HYVET-COG study, no reduced incidence of dementia was found in persons aged 80 years or older when treated with antihypertensive medication. However, when combining these data with results from other trials studying the effect of antihypertensive medication, the incidence of dementia was significantly lower in the treatment group [10]. In the SPRINT MIND trial, strict blood pressure (BP) regulation in cognitively healthy persons reduced the risk of mild cognitive impairment and the combined rate of mild cognitive impairment and dementia [11]. Along with the beneficial effects on the incidence of MACE, these findings have led to increasingly aggressive targets for BP control [12]. With regard to lipid-lowering therapy, in older persons with pre-existing ASCVD (i.e., secondary prevention), statins have been shown to be beneficial in reducing the risk of MACE. However, whether lipid-lowering therapy can prevent or delay MACE in older persons without CVD (i.e., primary prevention) remains unclear.

Persons with dementia were excluded from trials regarding BP and lipid-lowering therapy, while this may be a group of special interest when it comes to prevention of ASCVD for several reasons. Firstly, they often have a high risk of ASCVD. On the other hand, in this population, beneficial and harmful effects of CVRM may be less favorable compared to older persons without cognitive impairment. For patients with comorbidities, current international guidelines on CVRM recommend a more patient-centered approach, often leading to a less strict treatment regimen [13]. Whether these comorbidities also include dementia is not discussed.

Currently, due to multimorbidity, the majority of older persons with dementia are treated by several medical specialists, who may not all have specific expertise in this field. For these physicians, it can be challenging how to take into account dementia in the treatment plan, since guidelines are missing for this vulnerable patient group. To meet these requirements, we set out whether CVRM in persons with dementia calls for an approach that differs from the guidelines. We created an overview of the recommendations on CVRM in current guidelines, evidence from randomized and observational studies, aspects to consider when estimating the beneficial and harmful effects of CVRM in persons with dementia, and when to consider deprescribing. We aim to provide clinicians with tools on when to start, stop or intensify BP- and lipid-lowering treatment, and how to evaluate therapy in these patients.

## BLOOD PRESSURE AND LIPID TARGETS FOR OLDER PERSONS WITH COMORBIDITIES ACCORDING TO CURRENT GUIDELINES

In current CVRM guidelines, given BP and lipid targets are based on medical history (history of ASCVD) and cardiovascular risk (risk of MACE). Age, comorbidities, and frailty are also taken into account when determining to what extent BP and lipids should be lowered. However, frailty definitions and more specific information about these comorbidities are missing in guidelines. In Tables 1 and 2, we created an overview of the recommendations on BP targets and lipid-lowering treatment for older persons and persons with comorbidities according to the (inter)national guidelines for CVRM in adults. None of these guidelines provided recommendations specifically for persons with dementia.

**Table 1:** Recommendations in (inter)national guidelines regarding blood pressure targets. SBP, systolic blood pressure; DBP, diastolic blood pressure

Guideline	General recommendations in older persons	Recommendations in very old or in the event of comorbidity
WHO 2021 [14]	No recommendations specifically for older persons.	Comorbidities and older age are not mentioned.
American College of Cardiology/American Heart Association 2017 [15]	‘SBP target < 130 mmHg for noninstitutionalized ambulatory community-dwelling persons≥65 years of age.’	‘In the event of a high burden of comorbidity and limited life expectancy, clinical judgement, patient preference, and a team-based approach to assess risk/benefit is reasonable for decisions regarding intensity of BP lowering.’
		High burden of comorbidity/ limited life expectancy/ clinical judgement are not further specified.
European Society of Cardiology/European Society of Hypertension 2018 [12]	‘SBP target 130–139 mmHg, DBP target < 80 mmHg if tolerated for persons≥65 years of age.’	‘For persons > 80 years of age who have not yet received treatment for BP, treatment is recommended when their SBP is≥160 mmHg, as long as treatment is tolerated.’
		No recommendations for comorbidities.
National Institute for Health and Care Excellence 2019 [16]	‘SBP target < 140 mmHg and DBP target < 90 mmHg for adults < 80 years of age.’	‘SBP target < 150 mmHg and DBP target < 90 mmHg for adults≥80 years of age.’
		‘In the event of frailty or multimorbidity: use clinical judgement.’
		Frailty/multimorbidity/clinical judgement are not further specified.
Dutch guideline on CVRM in older persons 2019 [17]	‘SBP target < 150 mmHg and if tolerated < 140 mmHg, for adults≥70 years of age without physical frailty.’	‘SBP target < 150 mmHg, as long as DBP stays > 70 mmHg for adults≥70 years of age with physical frailty.’
		Physical frailty is not further specified.

**Table 2:** Recommendations in (inter)national guidelines regarding the initiation and continuation of lipid-lowering treatment. CVD, cardiovascular disease; SBP, systolic blood pressure; DBP, diastolic blood pressure

Guideline	General recommendations in older persons	Recommendations in very old or in the event of comorbidity
American College of Cardiology/American Heart Association 2018 [18]	‘Lipid-lowering treatment is recommended for primary prevention according to level of risk in persons≤75 years of age.’	‘Lipid-lowering treatment for primary prevention in persons > 75 years of age is based on clinical assessment and can be stopped in the event of functional decline, multimorbidity, frailty or reduced life-expectancy.’
‘Lipid-lowering treatment is recommended for secondary prevention in persons≤75 years of age.’	‘Lipid-lowering treatment for secondary prevention in persons > 75 years of age is reasonable after evaluation of the potential risk reduction, patient frailty and preference.’
		Frailty is not further specified.
European Society of Cardiology/European Atherosclerosis Society 2019 [19]	‘Lipid-lowering treatment is recommended for primary prevention, according to the level of risk, in older persons≤75 years of age.’	‘Initiation of lipid-lowering treatment for primary prevention in persons > 75 years of age may be considered if at high or very high CVD risk.’
	‘Lipid-lowering treatment is recommended for secondary prevention for persons≥65 years of age.’	No recommendations for comorbidities and physical frailty.
National Institute for Health and Care Excellence 2014	No general recommendations based on age	‘Take into account comorbidities, general frailty, and life expectancy.’
		Comorbidities/general frailty/life expectancy are not further specified.
Dutch guideline on CVRM in older persons 2019 [20]	‘Consider lipid-lowering treatment for persons≥70 years of age with extremely elevated CV risk for primary prevention.’	‘Do not start lipid-lowering treatment for persons with physical frailty for primary prevention.’
		‘Consider lipid-lowering treatment in persons with physical frailty and sufficient life expectancy for secondary prevention.’
		Physical frailty is not further specified.

## EVIDENCE FROM RANDOMIZED AND OBSERVATIONAL TRIALS

### Antihypertensive treatment

To the best of our knowledge, up until now, there are no randomized controlled trials (RCTs) evaluating the effect of lowering BP on the prevention of MACE or cognitive decline in persons with dementia. Longitudinal studies regarding treatment of hypertension in persons with dementia show inconsistent results. In one study in persons with dementia and hypertension, better cognitive functioning was found in persons treated with antihypertensive therapy [21]. Yet, another observational study in older persons with dementia who received antihypertensive treatment showed that low daytime systolic blood pressure (≤128 mmHg) was associated with more cognitive decline compared to a higher systolic blood pressure (129–144 and≥145 mmHg) [22]. This suggests that lowering BP to a certain level may even deteriorate cognition. However, since the design of these studies are observational, confounding by indication could have explained at least a part of the effect, limiting the interpretation of the results. To the best of our knowledge, no observational studies exist on CVRM and the prevention of MACE in persons with dementia.

### Lipid-lowering treatment

There are currently no RCTs evaluating the effect of lipid-lowering treatment on the prevention of MACE or cognitive decline in persons with dementia. However, several observational studies in persons with dementia have shown an association between lipid-lowering treatment and cognitive function. Two longitudinal studies in persons with AD found that treatment with lipid-lowering therapy was associated with slower cognitive decline compared to persons without therapy [23, 24]. Also, an observational study evaluating the effect of treatment of vascular risk factors in persons with AD found slower cognitive decline when dyslipidemia was treated [25]. No data were reported regarding tolerance or side effects. These findings suggest a potential protective effect of lipid-lowering therapy in cognitive decline; however, the observational design of these studies limits the interpretation due to confounding by indication.

## ASPECTS TO CONSIDER WHEN ESTIMATING THE BENEFICIAL EFFECTS OF CVRM IN OLDER PERSONS WITH DEMENTIA

### Time to benefit and life expectancy

CVRM is mainly initiated to prevent ASCVD on the long(er) term. When considering preventive therapy, one should take into account when to expect the intended benefit (i.e., time to benefit) and life expectancy [26]. Survival time is significantly shorter in persons with dementia compared to the general population, which is most pronounced in young-onset dementia patients [27]. Among dementia subtypes, little variation in survival time has been observed. Median survival time after diagnosis is 6 years [27]. Especially severity of dementia, which can be classified as mild, moderate, or severe (also referred to as early, middle, and late), is a strong predictor of reduced survival, with a survival of only 1.4–2.4 years for severe dementia [28]. From the PROSPER-trial we know that in older persons with CVD and/or high cardiovascular risk, the time to benefit for statins to prevent MACE is approximately three years [29]. With regard to BP lowering the time to benefit is shorter; it takes up to one year of intensive BP treatment to prevent MACE [30]. Since persons with dementia have a reduced life expectancy, they may not live long enough to experience a (new) cardiovascular event. In other words, the time to benefit for treatment to reduce CVD risk may exceed the patient’s remaining life expectancy, especially in the event of severe dementia.

### Competing mortality risks and CVD risk calculators

For both patients with and without CVD, there are risk assessment tools available that calculate a 5- and 10-year risk of CVD [13]. These models also calculate the risk reduction when intensifying BP treatment and lipid-lowering treatment. However, not all these models account for life-expectancy, dementia, and frailty as competing risks. Especially, in persons with dementia, the competing risk of death is high as they have a high risk of dying from the complications of the dementia itself instead of dying from MACE [31]. Consequently, these risk scores tend to overestimate the actual 5- and 10-year risk of CVD and benefit from CVRM in the prevention of CVD in persons with dementia. They should therefore be used with care.

## ASPECTS TO CONSIDER WHEN ESTIMATING THE ADVERSE EFFECTS OF CVRM IN OLDER PERSONS WITH DEMENTIA

### Natural course of BP and lipids

In the decade before death, systolic blood pressure (SBP) and diastolic blood pressure (DBP) decrease as part of the natural course of aging. These BP decreases are most pronounced in persons with dementia, heart failure and late-life weight loss. In the very old (those dying at 90 year or older), SBP decreases even with more than 20 mmHg throughout the last decade [32]. In persons with dementia, BP starts to decrease prior to clinically apparent dementia and continues to decline afterwards [33–35]. This induces the risk of hypotension in persons with dementia who are on BP treatment when BP is not regularly checked. Therefore, after treatment is initiated and targets are achieved, BP should be monitored at least once a year (or more often in line with national guidelines) or sooner in the event of complaints. BP measurement is preferably performed in an ambulant setting in order to get insight in white-coat hypertension and large BP fluctuations, for example during a 24-hour measurement. When 24-hour measurement is not possible or eligible (for instance, in moderate/advance stage dementia, when it leads to confusion or agitation), we recommend repeated BP measurements. For example, depending on feasibility for clinician and patient: 1) morning and evening BP measurements at home during 3–5 consecutive days, taking two measurements on each occasion with an interval of 1 minute [36]; 2) an automatic measurement of 3 or more readings at the hospital or general practitioner’s office, without the attendance of a healthcare professional [37]; 3) repeated measurements during at least 2–3 office visits at 1–4 week intervals [37].

With regard to lipids in older persons there is a U-shaped association between cholesterol levels and mortality risk [38]. Higher cardiovascular and all-cause mortality risks in persons with low cholesterol levels are seen in those who are physical frail, suggesting that low cholesterol levels and mortality is caused by a third factor, such as comorbidities [39]. Interpretation of lipids in physical frail persons therefore requires clinical judgement. We suggest that lipid screening should be performed at least once after initiation of treatment.

### Orthostatic hypotension and diastolic hypotension

Compared to cognitive healthy individuals, persons with dementia are more likely to suffer from orthostatic hypotension (OH), which is particularly true for atypical Parkinsonian disorders such as Lewy body dementia and to a lesser extent for AD and VaD [40]. In addition, due to treatment of systolic hypertension, diastolic hypotension is often induced, particularly in older persons with coronary artery disease and diabetes mellitus. Diastolic hypotension and OH have been related to an increased risk of cardiac events, mortality, falling, and cognitive decline [41–44]. Moreover, OH is a significant predictor of the conversion of mild cognitive impairment to dementia [40, 45]. The hypotension/hypoperfusion damage hypothesis states that episodes of cerebral hypoperfusion lead to small-vessel disease, neurodegeneration, and progression of dementia [46]. It is therefore possible that excessive BP lowering in persons with dementia worsens cognition through cerebral hypoperfusion. However, it remains unclear at which BP level the harms of low DBP outweigh the benefits of lowering SBP [47]. Since persons with dementia are more likely to suffer from OH, BP should be measured supine (after minimum of 5 minutes of bed rest) and thereafter repeatedly upon standing, up to 3–5 minutes, at 1 minute intervals or continuously [48]. Medication should be adjusted when OH is diagnosed.

### Risk of adverse drug events

Polypharmacy is common among persons with dementia [49]. As a result of the cognitive impairment itself and drug-drug interactions, persons with dementia are at high risk of adverse drug reactions [50, 51]. Especially cardiovascular medication (ACE-inhibitors, alpha and beta blockers), psychotropic and analgesic drugs are associated with (preventable) adverse drug events such as falls [52]. With regard to statin treatment, one should be aware of drug-drug interactions that interfere with the metabolism and therefore elimination of statin, thereby increasing risk of adverse events such as myopathy. In persons with dementia, adverse drugs events are probably underreported due to memory bias and under-recognition due to unspecific signs [51, 53]. Persons with dementia should therefore be closely monitored for adverse drug reactions by their treating clinician.

### Patient’s preference and advance care planning

In order to improve quality of life of persons with dementia, treating clinicians should be aware of the patient’s treatment wishes and concerns about future health issues. These topics should be preferably discussed in the early stages of dementia, when the patient is still able to reflect on his situation. Advance care planning (ACP) is a continuous process of reflection and dialogue between the patient, those close to him and the treating physician, concerning the patient’s preferences and values when it comes to future treatment and care [54]. In the light of CVRM, these ACP conversations should focus on the patient’s treatment goals (improving survival or quality of life) and the perspective on taking preventive medication for risk reduction of MACE. The American Society of Clinical Oncology provides recommendations on how to guide these conversations with patients and their caregivers, which are also applicable to non-cancer advanced terminal illnesses [55]. When treatment goals are set, the treatment plan should be made by shared decision making [56].

## DEPRESCRIBING

Deprescribing is the process of withdrawal or dose reduction of an inappropriate medication, supervised by a health care professional with the goal of managing polypharmacy and improving outcomes [57]. In persons with limited life expectancy such as in persons with moderate or severe dementia, the life-time benefit of starting or intensifying CVRM is most likely of limited added value and therefore deprescribing should be part of the clinical decision process. In addition to time to benefit, aspects to take into account when deprescribing in persons with dementia are the natural course of BP, polypharmacy, interactions, side-effects and patient’s preference.

Data from RCTs in older persons aimed at deprescribing cardiovascular drugs are scarce. What we do know is that the majority of older persons using medication for the prevention of MACE, are willing to stop medication when their doctor says it is safe [58]. However, it is not always easy for clinicians to identify which older person is eligible for deprescribing cardiovascular medication. They may have concerns about interfering with other clinicians’ treatment plans, patients’ reluctance toward deprescribing or lack of patient understanding of deprescribing [59]. Only one RCT evaluated deprescribing of antihypertensive medication in persons with mild cognitive impairment [60]. Discontinuation did not improve cognitive functioning and was not related to MACE. However, follow-up time (16 weeks) was too short to assess long-term risks of discontinuation. A generalized statement about discontinuing all antihypertensive agents in older persons is therefore considered contentious [61]. Taking this into consideration, antihypertensive treatment in persons with dementia should at all-time be deprescribed in the event of intolerance or an adverse event. When deprescribing antihypertensive treatment, several guidelines can assist in when and how to deprescribe [62, 63]. Medications should be withdrawn one at a time, at 4-week intervals [64]. One should be aware of potential adverse events associated with drug withdrawal, such as palpitations after withdrawal of heart rate-limiting drugs or accelerated hypertension. After every withdrawal, BP should be monitored to ensure it remains on target [64]. With regard to statin discontinuation, there is one RCT in persons with a life expectancy≤1 year that showed no difference in the proportion of deaths between groups discontinuing and continuing statins [65]. This has led to the recommendation that lipid-lowering therapies should be discontinued in persons with a limited life expectancy (≤1 year) or severe physical or cognitive impairment (severe dementia) [61].

## CONCLUSION AND RECOMMENDATIONS FOR CVRM IN PERSONS WITH DEMENTIA

Up until now, there are no studies investigating the effect of CVRM in the prevention of MACE in persons with dementia. With regard to cognitive preservation, it remains unclear to what extent CVRM is beneficial or harmful in persons with dementia.

In order to prevent MACE, current guidelines provide extensive recommendations for BP and lipid-lowering treatment. Despite the increasing burden of dementia worldwide, these guidelines do not take into account the presence and severity of dementia. In our opinion, this is an important gap of knowledge, as considering CVRM is challenging in persons with dementia for multiple reasons. Firstly, especially persons with severe dementia have reduced life expectancy due to the competing mortality risk of the dementia itself and may not live long enough to benefit from CVRM treatment. This is especially the case for lipid-lowering treatment. Secondly, in persons with early stage dementia, life expectancy can be sufficient to start or intensify CVRM, although there is still a lack of evidence on benefits of lipid-lowering therapy for primary prevention in older persons (with and without dementia). Furthermore, persons with dementia are at increased risk of CVRM induced harm, such as overtreatment and (orthostatic) hypotension due to the natural course of BP in these persons. Finally, shared decision making with the patient can be challenging due to the cognitive impairment.

**Fig. 1:**
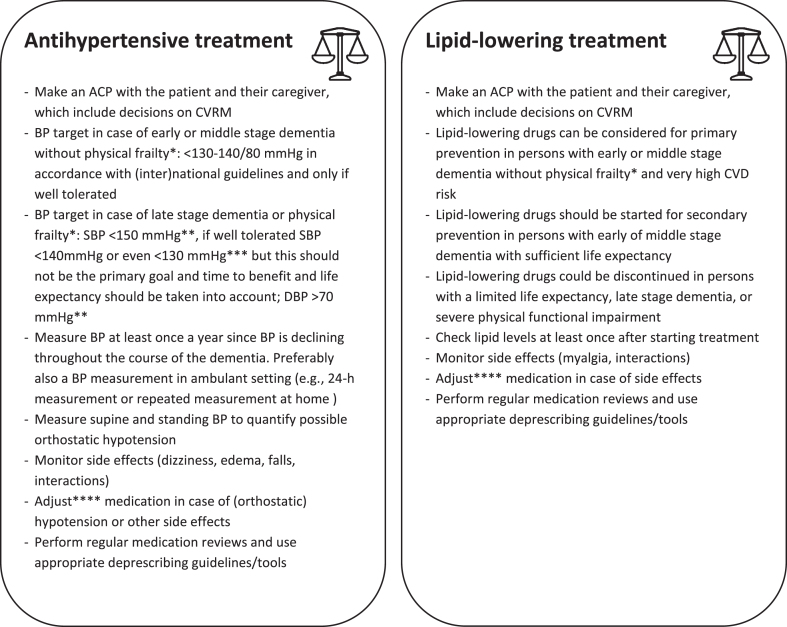
Practical guidance for CardioVascular Risk Management in persons with dementia. ^*^Physical frailty defined as low gait speed (< 0.8 m/s). ^**^According to Dutch guideline [17]. ^***^According to American College of Cardiology (ACC) / American Heart Association (AHH) [15]. ^****^Lower dose/switch/stop. ACP, advance care plan; CVRM, cardiovascular risk management; BP, blood pressure; SBP, systolic blood pressure; DBP, diastolic blood pressure; CVD, cardiovascular disease.

With regard to initiating, intensifying or withdrawing CVRM in persons with dementia, we formulated several recommendations, as summarized in Fig. 1. First of all, physicians should explore patients’ treatment wishes and take time for ACP. Then, they should take into account the severity of dementia and physical frailty of the individual patient for the aforementioned reasons. We suggest to use low gait speed (< 0.8 m/s) as an indicator for physical frailty since it is relatively simple to measure and associated with multiple adverse outcomes in older persons [66]. Next, traditional CVD risk calculators should be used with caution when estimating the beneficial effects of CVRM in persons with advanced stages of dementia, as they overestimate beneficial effects of CVRM. Furthermore, if BP treatment is initiated and/or intensified, one should ‘start low and go slow’. In other words, drugs should be started at the lowest effective dose, after which the dose is titrated to reach target BP levels. BP should be monitored at least once a year and medication reviews should be performed to check for drug-drug interactions. When adverse drug effects or intolerance occur, medication should be switched or withdrawn. In general, in persons with a limited life expectancy, deprescribing should be part of usual care.

## RECOMMENDATIONS FOR FUTURE RESEARCH

Ideally, future research should specifically focus on the effects of CVRM in persons with dementia. Research is needed to investigate the effect of CVRM on MACE in persons with dementia and on cognitive decline in the light of remaining life expectancy and adverse drug events. Observational studies are a good place to start, but we also realize that, due to the heterogeneity of this patient population, conducting RCT will be a challenge. Novel study designs, for example personalized risk models based on machine learning, may offer a solution.
